# The SAFIR project: an innovative high rate preclinical PET/MR detector towards dynamic multimodal imaging

**DOI:** 10.1186/2197-7364-2-S1-A15

**Published:** 2015-05-18

**Authors:** Robert Becker, Chiara Casella, Gunther Dissertori, Jannis Fischer, Alexander Howard, Astrik Jeitler, Werner Lustermann, Ulf Roeser, Qiulin Wang, Bruno Weber

**Affiliations:** ETH Zurich, Zurich, Switzerland; Tsinghua University Beijing, China; University of Zurich, Zurich, Switzerland

SAFIR (Small Animal Fast Insert for mRi) is an innovative, high rate, PET detector insert for MRI, to be used for quantitative dynamic pre-clinical imaging, with very high activities injected in the animals, up to 500 MBq. The PET detector will be designed to allow for ultra short acquisition periods (of the order of a few seconds) simultaneously with the MRI, permitting unprecedented temporal resolutions in preclinical dynamic multimodal imaging. High sensitivity (~ 6%), high spatial resolution (~1.5 mm FWHM), excellent coincidence timing resolution (CTR ~ 300 ps FWHM) and a fast DAQ system able to cope with the huge data throughput are required. Parallel with the hardware efforts, dedicated 4D algorithms for image reconstruction must be developed. The overall state of the project will be presented, including ongoing activities towards the choice and characterization of the detector components (crystals, SiPMs and readout chips), MonteCarlo simulations, and first reconstruction of various simulated sources. Special emphasis will be given to the results of a recent high rate test, where the TOFPET ASIC has been tested with Hamamatsu S12642-0404PB-50 SiPM arrays coupled to matrices of LYSO:Ce crystals (3.1x3.1x12 mm^3^ each), exposed to a 500 MBq activity of FDG radiotracer in a volume of about 0.5 cm^3^.

